# Supplementary material for: Fusaricidins, Polymyxins and Volatiles Produced by Paenibacillus polymyxa Strains DSM 32871 and M1
Source: Pathogens. 2021 Nov 15;10(11):1485. doi: 10.3390/pathogens10111485 (PMC8621861; doi:10.3390/pathogens10111485)
Supplement: Supplementary file 1 [file pathogens-10-01485-s001.zip › pathogens-1446860-supplementary.pdf]

Supplementary Materials

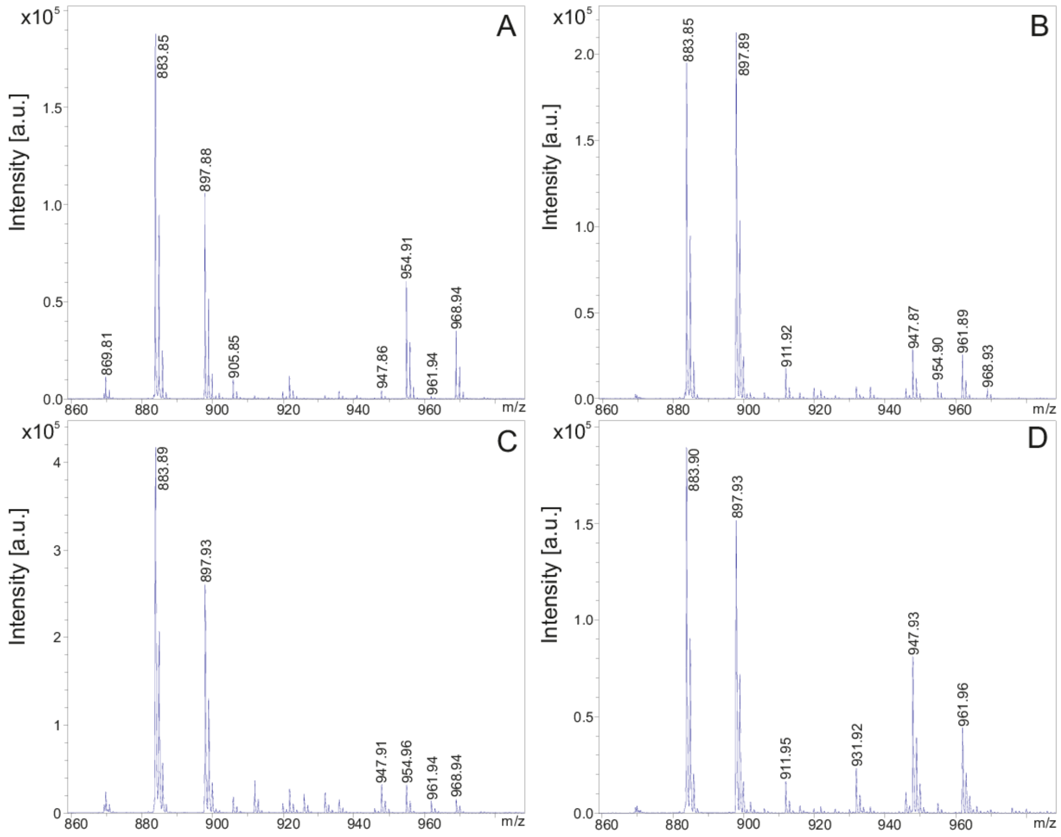

Figure S1. MALDI-TOF mass spectra of the fusaricidins in surface extracts and culture filtrates of *P. polymyxa* DSM 32871 (A and B) and M1 (C and D). For the preparation of surface extracts the strains were grown either on Landy or LB agar (A and C). Cultivation of the strains were performed either in the LOP- or LB medium (B and D) for 48 h.

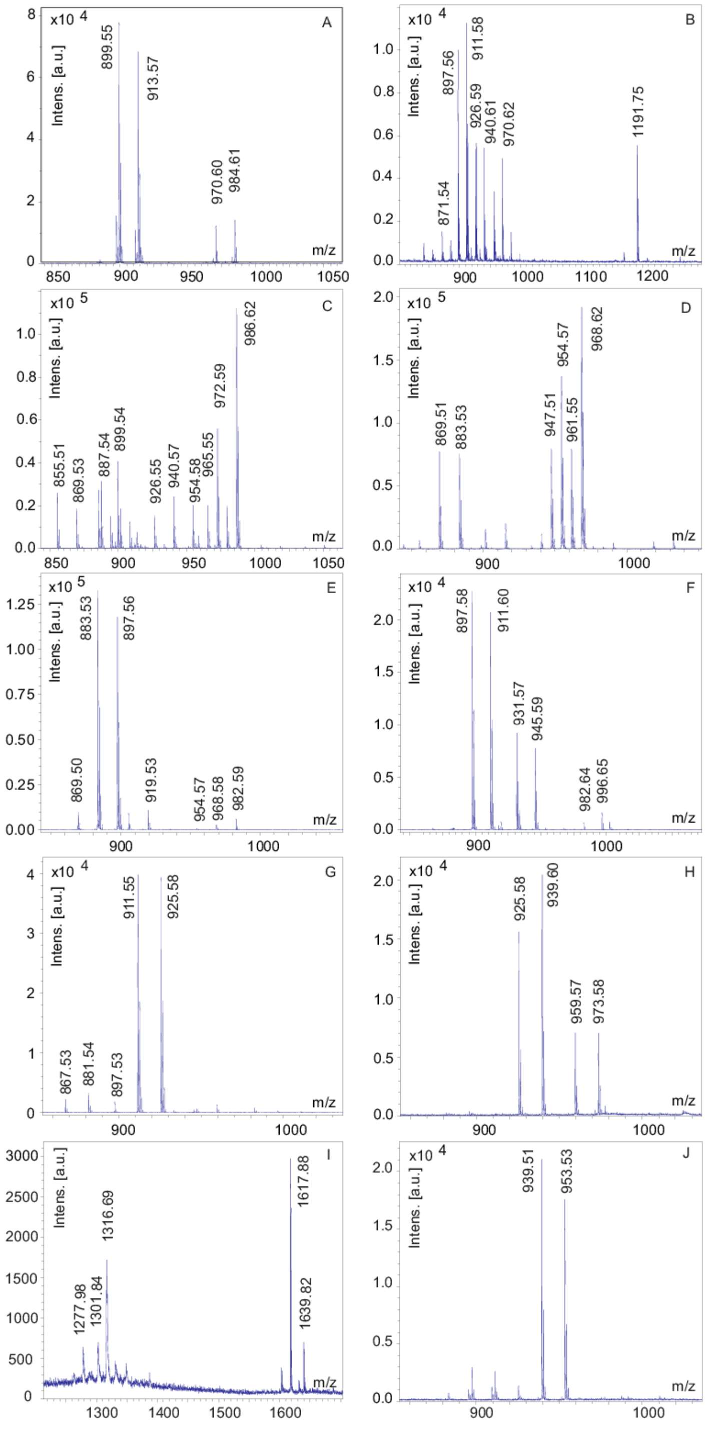

Figure S2. MALDI-TOF mass spectra of the products found in fractions 22 until 30 (A – J) of the HPLC-fractionation of the culture filtrate of DSM 32871 shown in Fig. 2B.

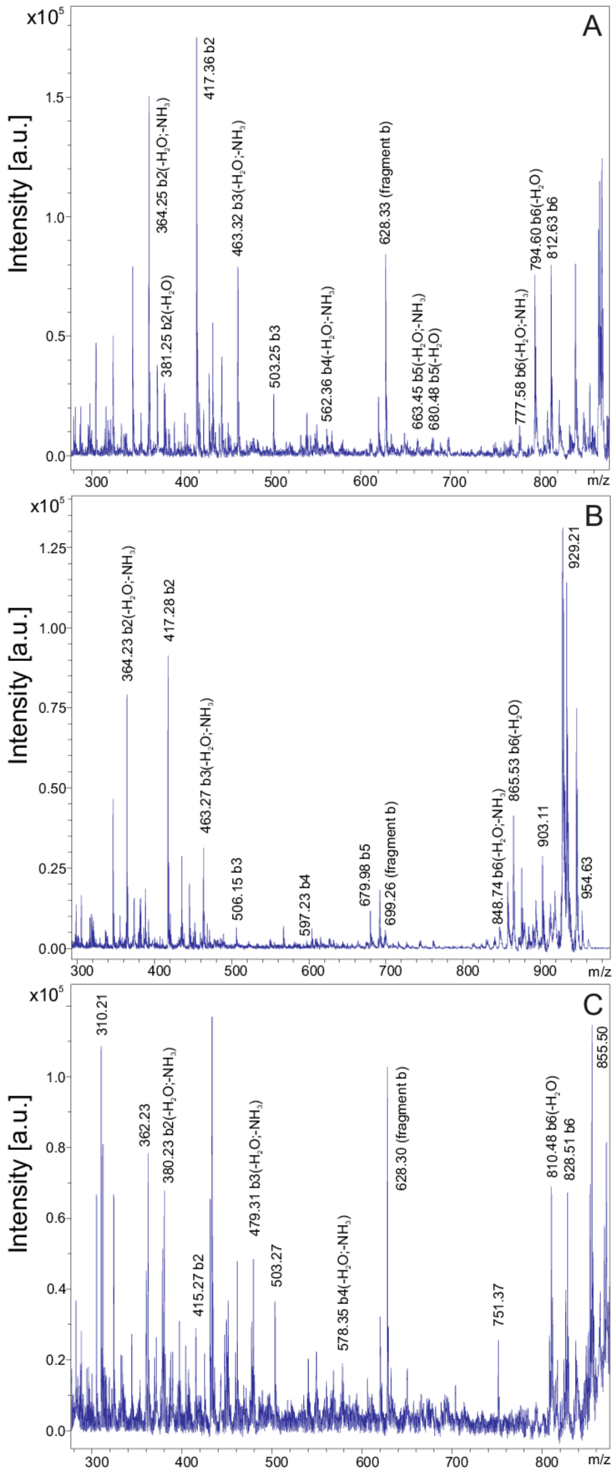

Figure S3. Upper part of the LIFT-MALDI-TOF/TOF product ion spectra of fusaricidin A ($[M + H]^+$ = 883.74) (A) and its derivatives with molecular masses $[M + H + 71]^+$ = 954.78 and $[M + H + 16]^+$ = 899.43, resp., in the range from 280 Da up to the parent ion.
